# Exploring resonance theory and uncontrollability during co‐creative art making: A qualitative study among cancer patients

**DOI:** 10.1002/cam4.7044

**Published:** 2024-03-28

**Authors:** Yvonne Weeseman, Michael Scherer‐Rath, Nirav Christophe, Henny Dörr, Esther Helmich, Mirjam A. G. Sprangers, Niels van Poecke, Hanneke W. M. van Laarhoven

**Affiliations:** ^1^ Department of Medical Oncology Amsterdam University Medical Centers, University of Amsterdam Amsterdam The Netherlands; ^2^ Cancer Center Amsterdam, Treatment and Quality of Life Amsterdam The Netherlands; ^3^ Faculty of Philosophy, Theology and Religious Studies Radboud University Nijmegen The Netherlands; ^4^ HKU University of the Arts Utrecht Utrecht The Netherlands; ^5^ Amsta Healthcare Organization Amsterdam The Netherlands; ^6^ Medical Psychology Amsterdam UMC location, University of Amsterdam Amsterdam The Netherlands; ^7^ Amsterdam Public Health, Mental Health Amsterdam The Netherlands

**Keywords:** co‐creation in art, experiences of contingency, palliative cancer patients, quality of life, resonance theory, uncontrollability

## Abstract

**Purpose:**

Co‐creation, characterised by artists and patients creating a joint work of art, may support patients with the integration of life events, such as living with cancer, into their life story. In the process of co‐creation, resonance relationships between patients, artists and material may evolve that support such integration. Using the framework of resonance theory, we aim to investigate if and how patients move through the three phases of resonance during a process of co‐creation and explore the role of uncontrollability in this process.

**Methods:**

Ten patients who received cancer treatment with palliative intent completed co‐creation processes, which were audio recorded. These recordings were imported in Atlas‐Ti and analysed by applying content analysis. We searched for the three phases of resonance, *Being affected*, *touched and moved*; *Self‐efficacy and responding*; *Adaptive transformation*. We additionally searched for signs of *uncontrollability*.

**Results:**

Patients used 4–8 sessions (median 5 sessions) with a duration 90–240 min per session (median duration 120 min). We found that patients move through the three phases of resonance during co‐creation processes. Uncontrollability both presents a challenge and an invitation to integrate experiences of contingency into one's life narrative. Patients express self‐recognition and the experience of contingency in their work of art.

**Conclusions:**

Integration of experiences of contingency into a life narrative can be supported by the process of co‐creation of art, which invites patients to relate to their illness, their environment and themselves. The phases of resonance in combination with uncontrollability as a continuously present factor, provide a means to both study and support the integration of experiences of contingency into the life narrative.

## Graphical abstract

In the process of co‐creation, resonance relationships between patients, artists and material may evolve that support integration of experiences of contingency. In the current study we aim to investigate, if and how, palliative cancer patients move through the three phases of resonance during a process of co‐creation and explore the role of uncontrollability in this process.

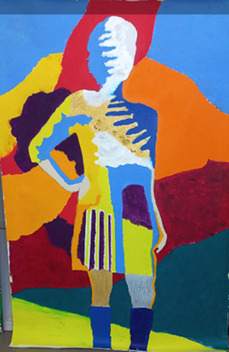

## 1 INTRODUCTION

The diagnosis of cancer in a palliative stage can be experienced as a life event that disrupts one's life narrative.^1^, ^2^, ^3^, ^4^, ^5^ Such a life event is neither impossible nor imperative; it could also not have happened; it is of a contingent nature. Life events that challenge one's ultimate life goals and worldview could evoke an experience of contingency affecting the fundamentals of one's own life, leading to an interpretation crisis.^6^, ^7^, ^8^ This crisis is often accompanied by questions like ‘why me’ and ‘why now’, and may prompt an individual to change one's life narrative.^1^, ^9^, ^10^ The integration of the experience of contingency into one's life narrative is hypothesised to be beneficial to one's sense of meaning and coherence, which could enhance quality of life.^1^, ^2^, ^3^

As part of the *In search of stories* (ISOS) project, we designed and investigated a multimodal narrative intervention aiming to support cancer patients in the palliative stage with the integration of experiences of contingency into their life narrative.^11^, ^12^, ^13^, ^14^ Within ISOS, participating patients are supported by professional artists and spiritual counsellors while they read selected literature,^11^ fill out life review questionnaires,^15^ draw rich pictures,^12^ go through a co‐creation process^14^ and reflect on these processes with the spiritual counsellor. During the co‐creation process with a professional artist, palliative cancer patients create a work of art while reflecting on their experiences of contingency.^13^, ^14^, ^16^

In previous research,^13^ we found that the integration of experiences of contingency showed resemblances with resonance theory, as described by Rosa.^17^, ^18^, ^19^ Resonance theory and contingency have previously been used as a theoretical framework in research on cancer patients, investigating a process of observing art works to create new meaning.^20^ In psychotherapy, when the focus is partly on retaining control through coping, uncertainty or uncontrollability can be seen as unfavourable.^21^ Contrary to this, Rosa describes ways for people to deal with uncontrollability without focusing on retaining control.^17^, ^18^ This could open new action perspectives to integrate experiences of contingency into the life narrative.

Resonance theory describes how an actual bi‐directional mode of relation can develop between people and the outside world, including others, when people are touched by those others and how they subsequently become changed by these so‐called resonance relationships. In a bi‐directional mode of relation the influence is reciprocal, i.e., one is influenced by other actors and vice versa.^17^, ^18^ The resonance process consists of three phases; 1. *Being affected*, *touched and moved*; 2. *Self‐efficacy*, *responding*, where one is able to respond to being touched and 3. *Adaptive transformation*, where a change in oneself due to engaging in the previous two phases can become visible and tangible.^17^, ^18^ Self‐recognition, i.e. to recognise oneself in the other, in that what touches you, is an important element of adaptive transformation.^17^, ^18^

An experience of contingency, such as living with cancer, could present a break with one's life narrative and could invite one to be receptive for the occurring life event. If cancer patients are able to be receptive to their new reality, they could connect with the experience of being ill, their environment and themselves. Then, bi‐directional modes of relation can develop in which patients' perspectives on the illness, their environment and themselves could change. Such modes of relation have the potential to facilitate the integration of both the experience of contingency and the changes introduced by the life event leading to the experience of contingency into one's life narrative.

Further, Rosa describes that uncontrollability is an important aspect of resonance, as resonance relationships only arise spontaneously and cannot be determined, controlled or forced. Uncontrollability can threaten one's desire for safety, but it can also surprise us and introduce possibilities for transformation.^17^, ^18^ Uncontrollability also forms an important element of contingency—things could have happened otherwise—as one has no control over how life develops. In co‐creation, the direction of the process of art‐making, in relation to the experience of contingency, cannot be completely determined, thereby also entailing an element of uncontrollability. For the current study, we define uncontrollability as “the experience of not knowing when and how the world is going to affect us, or not knowing beforehand how to relate to the experience. Unexpectedness is ingrained in the full experience of resonance”.^13^

The three phases of resonance theory could provide a theoretical framework to explain and understand how patients are receptive to the experience of contingency, encounter uncontrollability and subsequently transform themselves and their life narrative during co‐creation. An experience of contingency can leave patients searching for ways to describe and understand their interpretation crisis. ISOS aims to support palliative cancer patients in this process. Knowledge of the role and effect of contingency and resonance could support oncology and psychosocial clinicians in gaining a more comprehensive understanding of the challenges patients face, which could improve communication with patients.

Here, we aim to investigate if and how patients move through the three phases of resonance during a process of co‐creation and explore the role of uncontrollability in this process. We hypothesise that uncontrollability plays a prominent role both within the co‐creation process and during integration of experiences of contingency into one's life narrative.

## 2 METHODS

### 2.1 Participants

Palliative cancer patients participated in the ISOS project, which is a narrative multimodal intervention aimed at enhancing quality of life.^11^, ^13^, ^14^ ISOS is a joint project of the Department of Medical Oncology of Amsterdam University Medical Centres in collaboration with the Faculty of Philosophy, Theology and Religious Studies of the Radboud University, Nijmegen, the University of the Arts Utrecht and Amsta Healthcare Organisation Amsterdam. ISOS is funded by the Dutch Cancer Society, and participants were recruited from four Dutch hospitals; Medical Centres Amsterdam (AMC and VUMC), Spaarne Gasthuis Hospital and Maastricht University Medical Centre by their attending oncologist or nurse. Patients were offered no other psychosocial interventions. Further information is available on: https://cco.amsterdamumc.org/en/projecten/op‐zoek‐naar‐verhalen‐een‐onderzoek‐naar‐zingeving‐bij‐patienten‐met‐ongeneeslijke‐kanker/.

Participating patients were supported by professional artists and spiritual counsellors while they read selected literature,^11^ filled out life review questionnaires, drew rich pictures^12^ and embarked on a co‐creation process.^13^, ^14^ Patients who met the inclusion criteria were 18 years or older, in a palliative phase of cancer treatment, diagnosed with metastasized cancer less than 3 years prior to enrolment, had sufficient mental, verbal and physical capacity to participate, had a life expectancy of 6 months or more, which is beyond the planned duration of the ISOS intervention, including the co‐creation process with a professional artist and had provided written informed consent. Exclusion criteria were: a Karnofsky Performance Score <60 or a WHO score of 3, insufficient fluency in Dutch and a current psychiatric disease or cognitive impairment. Inclusion criteria for the current analysis were: participating in the ISOS project and having completed the co‐creation process.

In total, 11 professional artists participated in ISOS. These professional artists had previous experience with co‐creation with patients and were selected through the University of Arts Utrecht. Inclusion criteria for participation in the ISOS project were being able to work with multiple art modalities and having extensive experience in co‐creation processes. Prior to the start of the co‐creation processes, all artists attended a training of four sessions of 3 h each, focussing on how to work with palliative cancer patients.

### 2.2 Study design and data collection

We used a qualitative study design where all co‐creation sessions of 10 cancer patients within the ISOS project were audio recorded by the seven attending professional artists. The audio recordings were sent to the researchers for further analyses. In addition to the audio recordings, pictures taken of the work of art during various stages of the co‐creative processes were collected. YW translated the presented quotations from Dutch into English, which were endorsed by the co‐authors.

### 2.3 Data analyses

The current study uses an interpretative methodologic approach whereby resonance theory, emerging from sociology, fits a social constructivist point of view on analysing the data. We chose to use content analysis, which is a qualitative approach suitable to inductively research content, analyse its structure and establish main concepts.^22^, ^23^ We first used an approach of open coding to investigate what would emerge from the data. See Table 1 for the main procedural steps of our primary data analyses. Secondly, content analysis is also suitable to validate or extend an already existing theoretical framework and to search for already established concepts, called ‘category definitions’. After our initial inductive analysis, we used these codes in combination with existing literature^17^, ^18^, ^19^ to establish a category definition matrix for a deductive content analysis. The category definitions were based on resonance theory; 1. *Being affected*, *touched and moved*; 2. *Self‐efficacy*, *responding*; 3. *Adaptive transformation*; 4. *Uncontrollability* (see Table 2). The category definition matrix is refined during the coding process and allows for adding new categories when parts of the data do not fit the category definitions.

**TABLE 1:** Main procedural steps of the data analyses.

1. The audio recordings of the co‐creation sessions of 10 participating patients were imported in AtlasTi. 2. To familiarise herself with the data, YW extensively listened and re‐listened to the audio recordings. Before starting the coding process, emerging preliminary findings were discussed in detail with MSR and written down in a log. 3. YW started with an inductive coding process where all fragments were labelled with a code. Findings were extensively discussed with MSR and HvL. 4. To improve reliability and decrease bias, YW recoded sections of the data set. The recoding indicated satisfactory intra‐rater reliability. 5. Subsequently, a categorization matrix was developed that consisted of the categories ‘Being affected’, ‘Self‐efficacy’, ‘Adaptive transformation’ and ‘Uncontrollability’. To support construct validity, these main themes were compared with previously established themes of resonance from previous research and literature. All procedures were discussed with MSR and HvL. 6. YW then proceeded with a deductive coding process based on the created matrix. In this step, the codes created during the inductive coding were fit into the category matrix. The categorisation of the codes was discussed with MSR and HvL. 7. The category definitions ‘Being affected’, ‘Self‐efficacy’ and ‘Uncontrollability’ remained unchanged. ‘Adaptive transformation’ was divided into two new categories ‘Adaptive transformation – work of art’ and ‘Adaptive transformation – self recognition’ as this better fitted the data. The categorisation matrix was adapted accordingly, which resulted in all codes fitting the adapted categorisation matrix. All procedures and findings were discussed with MSR and HvL.

**TABLE 2:** Category definitions.^a^

Category definition	Descriptions
*1*. *Being affected*, *touched and moved*	The first moment of unexpectedness when the subject is affected by the world, in other words, is touched or moved in such a way as to develop an intrinsic interest in the segment of the world encountered and feels somehow addressed by it.
*2*. *Self‐efficacy*, *responding*	The affected person is challenged to actively react and reciprocate the call of that which moved him or her.
*3*. *Adaptive transformation*	A change in the person induced by experiencing the encounter with the unexpected. Being affected will add something new to the experience and can lead to a different perspective on the circumstances. The new perspective is intrinsically connected to existential values and experiences, but also generates new energy to welcome newness and different thoughts and feelings.
*4*. *Uncontrollability*	The experience of not knowing when and how the world is going to affect us, or not knowing beforehand how to relate to the experience. Unexpectedness is ingrained in the full experience of resonance

^a^ Category definitions based on Weeseman et al.^13^

The audio recordings of the co‐creation sessions were imported in AtlasTi^24^ and were primarily analysed by YW (MA, MSc, female), who has a professional background in art therapy, clinical psychology and spiritual care. All inductive and deductive analysis, the establishment of the category definitions and the results of all stages of the data analyses^24^ were extensively discussed with MSR (Associate Professor, PhD, male), who has a professional background in religious studies, theology, spiritual care and qualitative research and HvL (Professor, MD, PhD, female), who has a professional background in medical oncology and theology.

### 2.4 Ethics

The current study was carried out in accordance with relevant regulations and guidelines of the Declaration of Helsinki. As the Medical Research Involving Human Subjects Act was not applicable (reference number: W20_436 # 20.483), the study was exempted from ethical approval by the Medical Ethics Review Committee of the Amsterdam University Academic Medical Centres. All patients and artists have given written informed consent. The written informed consent included permission to use any data collected during the ISOS project for publications, including all audio recordings of sessions, pictures taken during the co‐creation sessions and pictures of the works of art.

## 3 RESULTS

### 3.1 Participants and data collection

At the time of the analysis of the current study, 10 patients had completed the co‐creation process and were hence included. In total, 25 patients were involved in the ISOS project. At the time of analyses, due to deteriorating health, four patients had not been able to finalise the co‐creation process. The other 11 patients were still participating in the co‐creation processes. The characteristics of these patients and the artists with whom the patients were partnered during the co‐creation process are shown in Table 3. There were no notable differences in age, gender and type of cancer diagnosis between the sample of the first 10 patients of the current study and the other 15 patients. In total, 35 patients have been invited to enrolment in ISOS of which 10 declined participation. Reasons for declining participation were fatigue, ongoing intensive treatment and lack of affinity with the modules of the ISOS intervention. As no specific data has been collected of these patients, we do not know if their demographics differ from those of the participants. The analysed co‐creation processes took place between March 2021 and August 2022, at the professional artist's studio, at the home of the patient or occasionally at another location, for example, a museum or a forest. The co‐creation processes involved four to eight sessions. Median number of sessions was five. The duration of the co‐creation sessions lasted between 90 and 240 min; median duration was 120 min.

**TABLE 3:** Participating patients, artists and patient‐artist dyads.^a^

Patients				Artists		
Number	Age	Gender	Cancer diagnosis	Expertise	Age	Gender
1	45	Female	Cervical cancer	Visual artist (A)	38	Female
2	71	Female	Cholangio cancer	Scenographer (B)	50	Female
3	57	Female	Bile duct cancer	Visual artist (A)	38	Female
4	59	Female	Colon cancer	Musician (C)	43	Male
5	66	Male	Oesophagus cancer	Musician (D)	45	Male
6	81	Male	Oesophagus cancer	Musician (D)	45	Male
7	55	Female	Pancreatic cancer	Scenographer (E)	50	Female
8	61	Female	Breast cancer	Visual artist (F)	64	Male
9	68	Female	Rectal cancer	Visual artist (G)	52	Female
10	64	Female	Neck cancer	Scenographer (B)	50	Female

^a^ Table after Weeseman et al.^16^

### 3.2 Phases of resonance and uncontrollability

In our analyses, we looked for (1) the presence of the three phases of resonance within co‐creation processes, (2) how patients moved through these phases and (3) the role of uncontrollability. For this study, we subdivided the third phase of resonance (*Adaptive transformation)* into two categories. During phase 3.1 (*Adaptive transformation: work of art*), the work of art is created and consolidated. Subsequently, during phase 3.2 (*Adaptive transformation: self‐recognition)*, the patients reflect on both the co‐creation process and the work of art, recognise themselves and transcend their former life narrative.

First, we will present the results of the first part of our research question, if patients move through the three phases of resonance during a process of co‐creation, by providing an overview of the presence of the three phases of resonance for all 10 patients. Then, we will explore the phases of resonance including the role of uncontrollability by providing a detailed description of the co‐creation process of one female patient.

#### 3.2.1 Phases of resonance in palliative cancer patients during co‐creation

The three phases of resonance were found in the co‐creation processes of all 10 patients. The phases of resonance are initiated when patients are touched and moved by pictures, drawings, colours, shapes, materials, or sounds that are presented to them. Subsequently, they responded by following their own impulses, creating various initial art expressions, such as plastering a mould of one's hands, making a flower compilation or creating a clay work in the shape of a cobra. Patients also responded to being touched by attempting to use more control to counter not knowing where an impulse would lead to. Patients alternated between these two responses. This is followed by adaptive transformation expressed in the work of art, for example, an arrangement of used bandages from chemotherapy treatment, which represent the recycling of life through various life cycles, or steel leaves, which represent a phoenix arising from the ashes of previous fears, while being supported by loved ones. Adaptive transformation is also visible in self‐recognition, as shown for example in the chosen colours representing the aura of a person leaving this world. Self‐recognition is particularly reflected in statements such as “I am free now and can play in a relaxed way”, or “I have become aware of what I have brought people, this was out of my awareness until now”. An overview of these processes and the phases of resonance for each patient individually is presented in Table 4.

**TABLE 4:** Overview of phases of resonance per individual patient.

Patient	1. Being affected, touched & moved	2. Self‐efficacy, responding	3.1 Adaptive transformation: work of art	3.2 Adaptive transformation: self‐recognition
1	Being touched by drawings of flowers, fresh and withered flowers, hands, being daddy's little girl and a cloud representing uncertainty.	Making a flower compilation resembling a uterus and letting it wither. Using the blanket as a locker to hide symbols and choosing a fitting print to project onto the blanket.	Projections of flowers in the shape of a uterus are embedded in a blanket. The blanket represents a nourishing and loving environment.	“The layering within the blanket represents the right for everything to exist. Each time when looking at it, something else becomes visible.”
2	Being touched by textile from several indigenous tribes, treatment bandage, turquoise colour, shapes of endlessness—the nautilus, fungi‐mycelium and natural materials like coral and shells.	Extending the life cycle of materials by recycling products. Wanting to create something to close a year of treatment. Connecting to nature. Answering the work of the artist by making her own work.	Used bandages from chemotherapy are re‐used to create works of arts that represent a closing of a period in her life and welcome the recycling of life.	“This is it, but here I am. Formulating my present life is important. Gratefulness for the time that I have left is reflected in the work of art”
3	Being touched by dreaming about curvy shapes, the upward movement and flexibility of sanseveria plants, the colours red and gold.	Creating a clay work in the shape of an upward cobra with a protective cloak and experimenting with making steel leaves representing the connection to people around me.	A corroded steel artwork of leaves representing a phoenix rising from previous fears and experiencing support. The corrosion represents the passing of time.	“Everything I am is embedded in this work of art. The way the wind of life blows is how the leaves whirl. I do not have to have a strong spine to withstand life.”
4	Being touched by the story of Orpheus and Euridice representing her and her husband, the music of Orpheus and walking in silence listening to sounds in nature.	Rewriting the story of Orpheus and Euridice to better fit her reality. Naming important nature elements during a silent walk. Selecting texts to add to the hiking map of the silent walk.	A hiking map that intends to lead to moments of reflection during the hike and ultimately leads to experiencing stillness as a state of being.	“Nature reflects the wonders of life. A tree can also have cancer but experiences it differently, just as it is. Nature encompasses presence, I notice that I now know I am a mortal being.”
5	Being touched by drawing a road with a heavy storm, a burning bush and light at the end of the road, the sound of an electrical guitar and sounds of thunder and motorbikes.	Wanting to create album art. Creating a new piece of guitar music in his own studio. Wanting to add letters of thanks to the album art and making a musical movie.	The journey of life is represented in a musical movie representing a road leading to the light while supported by loved ones.	“The blackness is here, but I am moving the other way. The false notes in the music make it realistic. It brings comfort, something tangible.”
6	Being touched by technical elements of the seraphine, listening to seraphine music and a fellow organist and choir playing in church and playing the toccata.	Wanting to create something with photography. Expressing what touches him in seraphine music and relating this to specific colours.	A musical movie with music played on the seraphine in a church that fulfils his need for meaning and connection.	“In working on this project I enter a world which I recognise but I find it hard to name. This is a reflection of me. I am free now and can play in a relaxed way.”
7	Being touched by the story of Orpheus and Euridice and listening to music inspired by the story, the shape of eggs, the colour white while painting an egg, bird feathers and Native American garments.	Felting a garment as a means to find inner stillness. Stitching feathers and horsehair onto the garment. Taking pictures of the process to create a booklet.	A white wool felt garment which is embroidered with bird feathers. The feathers represent a wider view on welcoming life and an attitude of softness.	“The felt craves the softness of the feathers. What is hidden in this is what I have carried throughout my life. When retrieving Euridice in my inner world, I trust that she will follow me towards love and harmony. Only through doing things, one finds out a different way.”
8	Being touched by portraits with bright colours and several shapes of status during a Renaissance exposition and exploring the use of colours in her favourite pictures of nature.	Posing for the artist. Thinking of words that fit her and colouring in drawings made by the artist.	A full body portrait representing experimenting with intense expression and passionate bright colours.	“The way I stand in this portrait is a statement, like a warrior. Thinking about working this way is new to me. It teaches me to see from a different perspective.”
9	Being touched by the story of the ‘Ant is gone’, recognising the impact the ant has had for the inhabitants of the woods, the images in books of Morandi and Katharina Grosse.	Drawing the eyes of the ant, as a reflection of her need to always wanting to notice everything. Drawing the shapes and colours of meaningful elements in life: dogs, pots and pans, an apron and her husband.	Multiple drawings representing a self‐portrait. The drawings contain aspects that are important to connect to in life.	“The way I have lived, withdrawn and open, is expressed in these drawings. It resembles connectedness. Looking at these drawings will support me in the time ahead.”
10	Being touched by reading the story of ‘The Metamorphosis’, selecting pictures for making a collage and sensing fabrics, her diaries and poem of a bridge about crossing over to the afterlife.	Looking for suitable pictures, plastering a mould of her hands. Re‐reading and shredding her diaries and creating paper mache out of them. Creating a paper mache bridge shape.	A bridge was created of paper from shredded dairies. The bridge represents a spiritual transition towards the afterlife, with love and support from loved ones.	“I did not come any further than what I had known, I needed this support to go beyond my own ideas. I have become aware of what I have brought people, this was out of my awareness until now.”

#### 3.2.2 How patients move through the phases of resonance and the role of uncontrollability

To illustrate how patients moved through the phases of resonance and experienced uncontrollability, we describe the co‐creation process of one patient, where the various aspects of the co‐creation process are most clearly visible in the interaction between patient, artist and material. Relevant quotations are presented and commented upon. In the comments, we indicate when uncontrollability or a phase of resonance emerges by inserting the characteristic in brackets.

**FIGURE 1:**
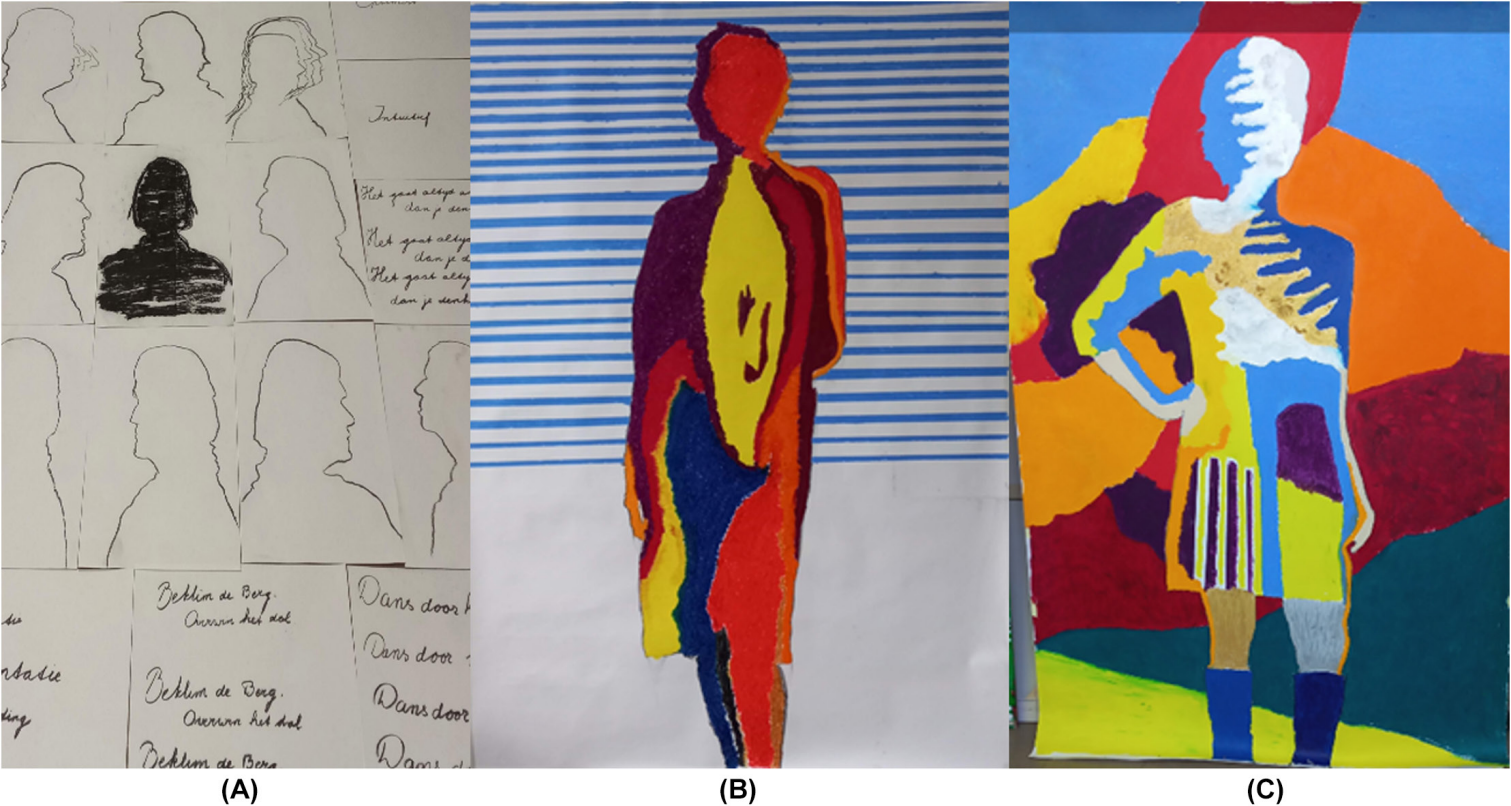
Paintings of the patient. From left to right: (A) initial drawings by the artist, (B) portrait 1, (C) portrait 2 (portrait 2 after Weeseman et al.^16^). (A) Initial set of upper body portraits made by the artist with words that describe the patient according to herself. (B) Portrait 1, drawing of the patient's whole body gestalt by the artist, coloured in by the patient, which the patient describes as “more elegant”. (C) Portrait 2, drawing of the patients gestalt by the artist, coloured in by the patient, which the patient describes as “tough, like a warrior, amazing how it fills up the whole frame”.

In patient 8, a 61‐year‐old female with breast cancer (see Table 3), her experience of contingency was connected to falling ill and subsequently feeling compelled to let go of her job, which she felt very responsible for and was very dedicated to. During the co‐creation process, she became aware of the sense of confinement she had experienced within her role as a team leader due to the responsibility she felt. She was challenged by the artist to move beyond her normal mode of being in control, where she first formulated plans before executing them. During co‐creation, she had to follow her impulses based on feelings, which made her adopt a new perspective on what she was capable of doing. It allowed her to discover her creativity and simultaneously invited her to challenge her perception of herself as a person who was used to refrain from following impulses. In Figure 1, a set of portraits of her made by the artist are presented. The latter two portraits are coloured in by the patient.

For their first session, the artist had taken the patient to a Renaissance exposition in the Rijksmuseum Amsterdam. Together they reflected on the portraits that distinctly spoke to the patient, illustrating receptivity (*Being affected*, *touched and moved*). These portraits were also linked to how she wanted to be viewed by others in her work, indicating a moment of self‐recognition.

*Patient: This lady has a real face*, *it is beautiful even though she is not actually looking at you*. *She does look pale and vulnerable*, *not much arrogance*. *And this pope is depicted in a real way*, *not serene but made out of flesh and blood*. *All these paintings show some kind of status sensitivity*. *I do feel that the way people dress says something about the way they are*. *As a team leader I like to wear grey*. *Maybe that is a kind of status symbol*.

Further in the co‐creation process, she is actively searching for a new way of self‐expression. The artist helps her by discussing possibilities. The patient shows receptivity for the input from the artist (*Being affected*, *touched and moved*), and also aspects of uncontrollability become visible, as she is becoming aware of a sense of uncertainty and not yet knowing what is going to be created (*Uncontrollability*). Yet she is receptive to this new experience, and she expresses hope that she will recognise the reflection of herself in the work of art.

*Artist: We can create an environment where we try to reach an abstract level*, *reaching beyond you*. *The starting point is your personality and the dialogue with your environment*. *So we could make something in which we get a sense of looking through your eyes*.

*Patient: So it is not a bad thing that I don't know what to do yet?*

*Artist: No not at all*. *Together we will create new ideas on what to make*. *That image will slowly come to the surface*. *We will find out what kind of colour schemes you like*.

*Patient: That would be completely your expertise*, *I can only sense instead of knowing what to do*. *It requires a very different way of thinking from what I am used to*. *For someone like me that is difficult as I am not used to this*, *I am more of a concrete thinker*, *not abstract*. *So I have to start thinking about what is important to me*, *whatever that may be*. *Well*, *my role is a difficult one*. *I hope I will recognise myself in the end result*.

In a subsequent session, the artist starts with the idea to draw a set of upper‐body portraits of the patient in one drawing and then to add some words the patient feels are describing her (Figure 1a). She responds by describing herself (*Self‐efficacy*, *responding*), and thus takes a new attempt to look for self‐identification and self‐reflection (*Adaptive transformation: self‐recognition*):

*Patient: I feel that acceptance*, *optimism*, *awareness and acquiescence are words that describe who I am*, *I wonder which word fits best*, *acceptance or acquiescence? Having the awareness that things always turn out differently than what one could have imagined also fits my attitude towards life*.

The artist continues by proposing to draw the patient's whole body gestalt (Figure 1b). This is somewhat uncomfortable for the patient as this is new to her, yet she is also receptive to this suggestion (*Being affected*, *touched and moved*). She is once again confronted with uncontrollability—a sense of uncertainty about what will unfold (*Uncontrollability*). She tries to counter the uncontrollability by proposing that the artist will tell her what to do instead of having to decide herself:

*Patient: This is so funny*, *how do you want me to pose? Should I look convinced*, *provocative or pompous? Or with a bit of vanity? You should tell me*, *Okay?*

*Artist: You can stand however you like*.

After the artist has made the drawings of the patient's gestalt, he invites her to fill in the drawings with oil pastel sticks. She is apprehensive to start, again confronted with uncontrollability as she feels uncertain (*Uncontrollability*), finding it difficult to overcome her initial fear to fail. She also becomes temporarily less receptive as she starts to feel blocked, which she gradually overcomes by accepting the challenge the painting presents to her (*Self‐efficacy*, *responding*):

*Artist: These are oil pastels*, *we will just start with colouring any given place*. *Where would you like to start?*

*Patient: I notice that I am starting to block myself*, *thinking in a difficult way*.

*Artist: When you just start*, *the next step will unfold naturally*. *Do you want to start?*

*Patient: Not really…*, *you said there is no way this can turn out to become ugly right? I would very much like to know what colour you want to see*. *Maybe it will become a bunch of bright colours*, *too bright*. *I would very much appreciate you leading the way*. *I know there is no right way*, *but still… I am thinking about which colour to start with*.

A myriad of options for colours to use are available, requiring openness to connect to what is beyond herself. She doubts, but eventually makes a choice and moves one step further in the process of creating something (*Self‐efficacy*, *responding*). In each session she is invited to work without a proposed plan (*Self‐efficacy*, *responding*), at the risk of doing it wrong (*Uncontrollability)*:

*Patient: I am not someone who is used to think in a vague manner*. *My deficiency*, *or maybe my habit*, *is that an initiative has to lead somewhere as if there is a plan from beginning to end*. *When I would do this type of work of art on my own I would probably get nowhere*, *but with you I am stimulated*. *I would still regret it if this will not turn out how I have it in mind*.

At each round of colouring she has to witness and endure her uncertainty of not being able to know (*Uncontrollability*) how to do the colouring in the right way (*Self‐efficacy*, *responding*). In both portraits, she is surprised by the end result. She reflects on colouring her first self‐portrait (Figure 1b), where we see self‐recognition and reflection on the evolving work of art (*Adaptive transformation: work of art and self‐recognition*):

*Patient: No one said this was going to be easy*. *It is like solving puzzles*, *playing chess and colourizing simultaneously*. *I really like it that we filled in small segments of the middle part*, *because now all the lines are connected*, *and all the colours are in two places simultaneously*. *Hey*, *when I take a few steps back the whole image looks different!*

During the co‐creation process, there is a constant interplay between receptivity to the input from the artist, the material and the new ways of thinking and working (*Being affected*, *touched and moved*). She is continuously answering the invitation of the artist to allow newness (*Being affected*, *touched and moved*) and to explore the use of bright colours (*Self‐efficacy*, *responding*). As illustrated below, she is making an effort to balance between spontaneity and strategic use of colours (*Self‐efficacy*, *responding*). Being confronted with the uncontrollability of the outcome of the artistic process, she expresses her expectations (*Adaptive transformation: self‐recognition*) and fear of ruining the work of art (*Adaptive transformation: work of art*). She also puts her fear of uncontrollability into words:

*Artist: How shall we approach the area around the portrait*. *Would you like to emphasize the colours*, *or shall we let the environment blend into the colours*. *Are we going to use primary or secondary colours*. *You could also think in terms of warm and cold colours*. *So when I place purple right here*, *the yellow will become even brighter*, *but if I use orange that piece of the portrait will radiate more warmth*.

*Patient: I think that when I now make a specific choice*, *I will have to use those colours consistently*, *and possibly the outlines of the figure will dissipate*. *I would find it unpleasant if I now choose a colour while you would have chosen something completely different*.

*Artist: To me all that matters is that you are going to make an associative*, *non‐strategic choice*.

*Patient: I find that really difficult because I think I might choose a colour that is not fitting*, *that would be unfortunate*, *it has to emanate intensity*, *and then I would ruin it*.

*Artist: It is oil paint so we can always paint a layer over this colour*. *The thing is that you are just going to do something*, *and then you take a few steps back and oversee what you've done*. *And then you do something and look again*. *It is a route you take each time again while you are painting*.

*Patient: Ok*, *so here we go again*, *I understand there are no limits*, *only me thinking within my own boundaries*. *I am normally thoughtful and don't react in a primary way*. *Being taken out of my comfort zone is a bit scary*.

In total, the patient and artist go through three rounds of the patient posing, the artist making various portraits of her gestalt, and the patient colouring in two of these portraits. Below, she reflects on the second portrait (Figure 1c) she has coloured in, expressing self‐recognition and reflection on the work of art (*Adaptive transformation: work of art and self‐recognition*):

*Patient: I think it has become amazing*, *it actually fills up the whole frame*. *It is definitely finished like this*. *All differently coloured areas*, *but it is also one coherent ensemble*. *Maybe I should take a picture of this one* (YW: she indeed did and used it for her social media account). *The first was more elegant*, *this one is tough*.

*If I would do another one*, *I would not colour it in like this*, *not completely even*. *I would use long lines*, *or maybe short stripes*. *As a whole it would look less fixed*, *more spacious*. *And I would use more pastel colours*. *I would like more variation*.

She further explains how she has experienced working on these portraits and how this experience relates to her thought processes concerning the experience of contingency, accentuating receptivity, self‐recognition and reflection in the work of art (*Being affected*, *touched and moved*, *Adaptive transformation: work of art and self‐recognition*):

*Patient: I find that the way I stand in the second portrait*, *on the right*, *is like a statement*, *it is like a warrior*. *I recognise myself in it*, *but the whole way of thinking and working is new to me*. *I never thought this way*. *It teaches me to look at things from a different perspective and simultaneously it is also a process in expressing more of myself and experiencing newness*.

During the co‐creation process, she looks back upon her life and on the co‐creation process (*Adaptive transformation: self‐recognition*). Up to the point of becoming ill, she has dedicated most of her time to her work, which involved responsibility in managing others. Below, she describes how, as time unfolds, she started to realise that she had put her own needs at the background and that she felt a need to change this, pointing to moments of self‐recognition and transformation (*Adaptive transformation: self‐recognition*):

*Patient: In the beginning of the intervention I have made a Rich Picture depicting what is most important to me*, *and drew a big heart in it*. *Within that heart I have written names of the people who are dear to me*. *Later I realized I have a good connection with my sisters and sons*, *and I find it very important that they are more frequently near me*. *It has become much more clear who I want to see and who I can refuse when people want to visit me*. *I used to find my work very important and was so honoured that at 61 I still got a new job as a senior manager*. *But that this job stopped because of my illness was in hindsight not bad*, *because I would have given myself away completely*. *It used to be so bitter to not be able to perform well at work*. *That would not be an option being a responsible manager*. *However*, *now I am starting to see that all those people at work just continue with their own lives without me*. *So now I make different decisions*.

This patient shows that her experience of contingency led her to be open for new perspectives during the co‐creation process, resulting in a change in perspective on her life narrative.

## 4 DISCUSSION

In this study with palliative cancer patients, we found that during co‐creation, a process of integration of experiences of contingency unfolds. This process is characterised by the phases of resonance, *Being affected*, *touched and moved*, *Self‐efficacy*, *responding*, *Adaptive transformation*, *work of art* and *Adaptive transformation*, *self‐recognition*.^17^, ^18^ All patients went through these phases of resonance. Uncontrollability forms an important factor in the process of integration of experiences of contingency.

Patients tended to start the co‐creation process with a desire to create something that reflects aspects of themselves, followed by being confronted with the uncontrollability of both the material and the process of art‐making. This confrontation could evoke a tendency to avoid uncontrollability, for instance, by trying to fall back on a previous idea of what to create or how to create it, or by trying to let the artist decide the course of the process. In this study, all patients, with help of the artists, overcame these struggles and were able to become receptive (again) in the face of uncontrollability.

During the co‐creation processes, ideas arose and were brought into materialisation, followed by the development of more ideas in the interaction with the material. These processes had an innate quality of asking the patients to be open to new experiences, which in turn enhanced their receptivity for new aspects of their thoughts, feelings and actions towards the experience of contingency on the one hand and self‐recognition in the created work of art on the other. As the created work of art reflected ‘new’ and ‘old’ aspects of their life narrative, patients recognised themselves both as familiar and as completely anew in the work of art. In the process of creating, new parts were added and others were left out or released. The inherent uncontrollability of the art making allowed for new experiences to arise and creates space for newness, probing receptivity in patients.^25^

The several stages of creating a work of art also reflected the transformation the patient has undergone in connection to the experience of contingency. The work of art formed a new starting point to view one's life narrative from a different perspective. As such, the works of art can be seen as a witness of the process a patient went through.^25^ Such a witness or work of art can serve as a tool showing what was hidden and remained secret, realising the full depth of the new reality.^25^ The work of art is a keeper of the experiences, expressed in different languages. As shown in Table 4, the language could pertain to words, a painting, a sculpture, a poem, a piece of music or a movie. Whatever language was used, the work of art materialised the experiences of contingency, which enabled patients to better understand and communicate their experiences and observations.^25^, ^26^

The integration of experiences of contingency into the life narrative during co‐creation shows resemblances to psychological resilience.^27^, ^28^ Psychological resilience, i.e. ‘the ability to cope successfully with external stress’^27^ is considered to be a major psychological protective factor supporting people to successfully deal with the distress caused by negative life events like a diagnosis of incurable cancer. Resilience allows patients not only to adapt to adverse circumstances but even to thrive despite these circumstances.^27^ In patients reflections on their works of art, we have seen how patients creatively adapt to the new reality of being in a palliative phase of treatment while taking part in a co‐creation process (see Table 4). A distinction between psychological resilience and our approach is that our study describes how patients, during co‐creation, create bi‐directional resonance relationships with essential aspects of their illness experience, including the uncontrollability and the limitations of the (undesired) new reality. Reflecting on aspects of their illness could provide an opportunity for patients to become aware of how they currently deal with uncontrollability in their lives and support them in finding balance between experiencing boundaries and exploring new creative opportunities arising from their illness experience.

A strength of the study is that we were able to closely and in depth follow the unfolding of the co‐creation processes. The audio recordings of the co‐creation sessions can be seen as the accounts of the consecutive steps patients took in the reflection on their experiences of contingency and the integration of these experiences into their life narrative. The artists and patients were not familiar with resonance theory. As such, there was no bias towards introducing the construct of resonance theory in the naturally unfolding co‐creation processes. The participating patients were not directly interviewed as we used the recordings of the co‐creation sessions. On the one hand, this could be a strength as participants were not led in any direction by specific questions. On the other hand, this might present a weakness as specific probing could have revealed deeper meanings of the process of art‐making and the integration of experiences of contingency into one's life narrative.

The use of an existing theoretical framework (resonance theory) that was evaluated for usefulness in two other studies allowed for a deductive analysis that could be performed efficiently and systematically. The deductive approach stimulated us to develop theoretical sensitivity and further enabled an in‐depth analysis of the co‐creation processes. However, a weakness of the study is that, as we used an existing theory, we could have induced positive bias. We tried to diminish this by discussing each step extensively and using a log, which served as a check‐up in the critical reflections held during the process of data analysis. The findings of the current study need to be confirmed by the future results of the remaining co‐creation processes to gain a more elaborate overview of how cancer patients could integrate experiences of contingency into their life narrative during co‐creation.

## 5 CONCLUSION

Integration of experiences of contingency into one's life narrative can be supported by the process of co‐creation of art, which invites patients to relate to their illness, their environment and themselves. The phases of resonance in combination with uncontrollability as a continuously present factor, provide a means to both study and support the integration of experiences of contingency into the life narrative.

## AUTHOR CONTRIBUTIONS

**Yvonne Weeseman:** Conceptualization (equal); formal analysis (equal); methodology (equal); project administration (equal); writing – original draft (equal). **Michael Scherer‐Rath:** Conceptualization (lead); formal analysis (equal); investigation (equal); methodology (lead); supervision (lead); validation (equal); writing – review and editing (equal). **Nirav Christophe:** Project administration (supporting); writing – review and editing (supporting). **Henny Dörr:** Project administration (supporting); writing – review and editing (supporting). **Esther Helmich:** Writing – review and editing (supporting). **Mirjam A. G. Sprangers:** Writing – review and editing (supporting). **Niels van Poecke:** Writing – review and editing (supporting). **Hanneke W. M. van Laarhoven:** Conceptualization (lead); formal analysis (equal); funding acquisition (lead); investigation (equal); methodology (equal); supervision (lead); validation (equal); writing – review and editing (lead).

## FUNDING INFORMATION

This study was funded by the Dutch Cancer Society, grant number 11507.

## CONFLICT OF INTEREST STATEMENT

The authors declare that they have no conflicts of interest.

## ETHICS STATEMENT

The current study was carried out in accordance with relevant regulations and guidelines of the Declaration of Helsinki. The study was exempted from ethical approval by the Medical Ethics Review Committee of the Amsterdam University Academic Medical Centres, since the Medical Research Involving Human Subjects Act was not applicable (reference number: W20_436 # 20.483). At the start of their enrolment written informed consent was obtained from every participating patient and artist.

## CONSENT FOR PUBLICATION

All patients and artists have given written informed consent. The written informed consent included permission to use any data collected during the ISOS project for publications, including all audio recordings of sessions, pictures taken during the co‐creation sessions and pictures of the works of art.

## Data Availability

Audio recordings are available upon request.
